# Brown Spider (*Loxosceles* genus) Venom Toxins: Tools for Biological Purposes

**DOI:** 10.3390/toxins3030309

**Published:** 2011-03-22

**Authors:** Olga Meiri Chaim, Dilza Trevisan-Silva, Daniele Chaves-Moreira, Ana Carolina M. Wille, Valéria Pereira Ferrer, Fernando Hitomi Matsubara, Oldemir Carlos Mangili, Rafael Bertoni da Silveira, Luiza Helena Gremski, Waldemiro Gremski, Andrea Senff-Ribeiro, Silvio Sanches Veiga

**Affiliations:** 1 Department of Cell Biology, Federal University of Paraná, CEP 81531-980 Curitiba, Paraná, Brazil; Email: olgachaim@ufpr.br (O.M.C.); dilzatrevisan@gmail.com (D.T.-S); dani_chaves@ufpr.br (D.C.-M); anacarolina.wille@yahoo.com.br (A.C.M.W.); valpf@ufpr.br (V.P.F.); fernando_matsubara@hotmail.com (F.H.M.); luiza_hg@yahoo.com.br (L.H.G.); senffribeiro@ufpr.br (A.S.-R); 2 Department of Structural, Molecular Biology and Genetics, State University of Ponta Grossa, CEP 84030-900 Ponta Grossa, Paraná, Brazil; Email: rafaelbertoni@uepg.br; 3 Pelé Pequeno Príncipe Research Institute, CEP 80250-060 Curitiba, Paraná, Brazil; Email: oldcar25@yahoo.com.br; 4 Catholic University of Paraná, Health and Biological Sciences Institute, CEP 80215-901 Curitiba, Paraná, Brazil; Email: w.gremski@pucpr.br

**Keywords:** *Loxosceles*, brown spider, venom, recombinant toxins, biotechnological applications

## Abstract

Venomous animals use their venoms as tools for defense or predation. These venoms are complex mixtures, mainly enriched of proteic toxins or peptides with several, and different, biological activities. In general, spider venom is rich in biologically active molecules that are useful in experimental protocols for pharmacology, biochemistry, cell biology and immunology, as well as putative tools for biotechnology and industries. Spider venoms have recently garnered much attention from several research groups worldwide. Brown spider (*Loxosceles *genus) venom is enriched in low molecular mass proteins (5–40 kDa). Although their venom is produced in minute volumes (a few microliters), and contain only tens of micrograms of protein, the use of techniques based on molecular biology and proteomic analysis has afforded rational projects in the area and permitted the discovery and identification of a great number of novel toxins. The brown spider phospholipase-D family is undoubtedly the most investigated and characterized, although other important toxins, such as low molecular mass insecticidal peptides, metalloproteases and hyaluronidases have also been identified and featured in literature. The molecular pathways of the action of these toxins have been reported and brought new insights in the field of biotechnology. Herein, we shall see how recent reports describing discoveries in the area of brown spider venom have expanded biotechnological uses of molecules identified in these venoms, with special emphasis on the construction of a cDNA library for venom glands, transcriptome analysis, proteomic projects, recombinant expression of different proteic toxins, and finally structural descriptions based on crystallography of toxins.

## 1. The Spiders of Genus *Loxosceles* and Loxoscelism

The spiders of the *Loxosceles *genus, commonly denoted as brown spiders, belong to the family *Sicariidae,* sub-order *Labidognatha*, order *Araneida*, class *Arachnida, *and phylo *Arthropoda* [1,2]. The Sicariidae family also comprises the spiders of *Sicarius *genus. Strong evidences show that the genera *Loxosceles *and *Sicarius *are old, having originated from a common sicariid ancestor and diversified on Western Gondwana, before the separation of the African and South American continents. Both sicariid genera are diverse in Africa and South/Central America. *Loxosceles *spiders are also distributed in North America and the West Indies, and have species described from Mediterranean Europe and China. Apparently African and South American *Sicarius *have a common ancestor and South African *Loxosceles *are derived from this group. New World *Loxosceles *also have a common ancestor and fossil data is consistent with the hypothesis of North America colonization by South American *Loxosceles via *a land bridge predating the modern Isthmus of Panama [3]. 

**Figure 1:**
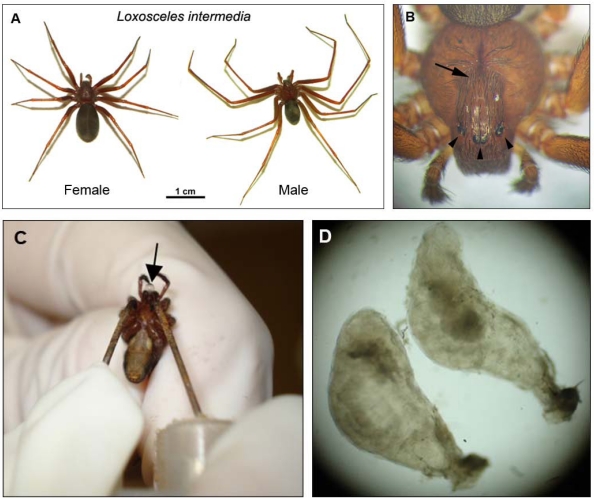
Brown spider aspects. (**A**) *Loxosceles intermedia *adult specimens—female and male. (**B**) Violin-shaped pattern (arrow) on the dorsal surface of cephalothorax from *Loxosceles intermedia* adult spider, and its six eyes arranged in pairs as a semi-circle (arrowheads). (**C**) Venom harvesting by electric shock applied to the cephalothorax. Arrow points for a drop of *Loxosceles intermedia* venom. Briefly, venom is extracted using an electric shock of 15 V applied to the cephalothorax of the spider and the venom from the tips of the fang is collected and diluted in phosphate buffered saline (PBS) or dried and stored at -80º C until use. (**D**) Brown spider venom glands of *Loxosceles intermedia* observed by stereo dissecting microscope (40X). Venom can be harvested directly from venom glands: the removed glands are washed in PBS and the venom is obtained by gentle compression of the glands.

The color of spiders of this genus ranges from a fawn to dark brown (Figure 1A). *Loxosceles* spiders have a violin-shaped pattern on the dorsal surface of their cephalothorax, vary in length from 1 cm to 5 cm, including legs, and have six eyes arranged in non-touching pairs in a U-shaped pattern (Figure 1B). This positioning of eyes has been described as the best means of identifying these brown spiders [4,5,6,7,8]. The brown spiders are sedentary, non-aggressive, have nocturnal habits and prefer to inhabit dark areas. In human habitats, brown spiders are often found behind furniture, pictures and associated with clothes.

Accidents involving *Loxosceles* genus spiders occur mainly in the warmest months of the year, predominantly during spring and summer [4,6]. The condition caused by brown spiders, categorized as Loxoscelism, is associated with a series of clinical symptoms including cutaneous lesions, which spread gravitationally from the spider bite. The lesions are characterized by necrotizing wounds that are dark blue-violet in color and become indurated, leading to the formation of scar tissue. Surrounding the lesion, there is also erythema and edema. At the systemic level (less frequent than the appearance of skin lesions), patients may experience fever, weakness, vomiting, pruritic reactions, renal failure, and hematologic disturbances that may include thrombocytopenia, disseminated intravascular coagulation and hemolytic anemia [5,6,8,9].

## 2. The *Loxosceles* Venoms

Over recent years, *Loxosceles *genus spider venoms have been studied by several scientific research groups worldwide, and many different toxins have been identified in the venoms. The corresponding biological and biochemical properties of these toxins have been reported, yielding insights into the pathophysiology of envenomation [4,5,7]. The venom of *Loxosceles* spiders is a complex mixture of protein and peptide toxins with a molecular mass profile ranging from 1 to 40 kDa [5]. To date, several molecules in the *Loxosceles* spider crude venoms have been described, including alkaline phosphatase [5,10], 5’-ribonucleotide phosphohydrolase [5], sulfated nucleosides [11], hyaluronidase [5,12,13,14], fosfolipases-D [5,15,16,17], metalloproteases, serine proteases [12,13,18,19,20,21,22] and insecticide toxins [23]. Table 1 contains a brief collection of main features from proteic toxins described in *Loxosceles* genus. 

Low molecular weight components, such as neurotoxic and non-neurotoxic peptides, polyamines and other components are poorly studied in *Loxosceles *venom. Using NMR-spectroscopy, Schroeder and colleagues (2008) showed that sulfated guanosine derivatives comprise the major small-mollecule components of the brown recluse spider. They detected cross-peaks corresponding to 2,5-disulfated guanosine and 2-sulfated guanosine. It appears that sulfated nucleosides occur in several spider superfamilies, such as Agelenoidea and Amaurobioidea. The physiological properties of the sulfated nucleosides remain largely unexplored [11].

Serine proteases were already described in *Loxosceles *venom as high molecular weight enzymes (85–95 kDa) with gelatinolytic activity activated by trypsin [19]. Proteome and transcriptome analyses of *Loxosceles *venom also described this family of proteases [24,25]. Serine proteases generally are among the best characterized venom enzymes affecting the hemostatic system. However, the exact role of serine proteases in envenomation still remains to be clarified.

Recently, by using a cDNA library and transcriptome analysis, a novel expression profile has been elaborated for *Loxosceles intermedia* gland venom. This recently developed profile has allowed the identification of additional toxins as components of the venom, including insecticidal peptides similar to knottins (molecules that form an inhibitor cystin knot), astacin-like metalloproteases, venom allergen, a translationally controlled tumor protein family member (TCTP), serine protease inhibitors, and neurotoxins similar to Magi 3 [26,27]. Brown spider venoms display a broad diversity of toxin isoforms, including members of the phospholipase-D family and astacin-like toxins, even in the same sample [17,27,28,29]. Such features, which represent an adaptation to increase the survival of the spiders and the effectiveness of venoms, confer advantages to the spider predator. To confirm the existence of a new family of toxin isoforms, it is necessary to further characterize their biological properties. Recently, a spider toxin database called Arachnoserver which was manually curated [30], has cataloged 54 toxins from Sicariidae spiders family. It was elaborated, based on information gleaned through studies on complex venom mixtures, and has resulted in an exponential increase in the identification of peptide-toxins. King *et al.* [31] recommend a rational nomenclature for naming toxins from spiders and other venomous animals to avoid the continued use of *ad hoc* naming schemes that introduce confusion and make it difficult to compare toxins among species and establish evolutionary relationships.

**Table 1:** An overview of toxin families in *Loxosceles *genus.

Toxins	MW (kDa)	Characteristics and actions described	No. Seq *
Phospholipases-D (SicTox family members, such as LiRecDTs)	30–35	Several isoforms with variant features such as: - Dermonecrosis [12,13,16,32,33,34,35,36,37,38] - Lipids hydrolysis [33,39,40,41,42] - Hemolysis [38,43,44,45] - *In vitro *platelet aggregation [34,36,37] - Infiltration of inflammatory cells [35,36,37,42] - Edema [34,38] - Renal disturbances [35,46] - Lethality [34,38,46,47] - *In vitro* cytotoxicity [35,42,46] - Cytokine activation [41,48,49,50]	335
Insecticidal peptides	5–8	- LiTx family members [23,27] and Magi 3-related peptides [23,27,51] - LiTx: Lethal to *S. frugiperda *(flaccid paralysis) [23] - LiTx3: appears to act upon Na^+ ^channels [23]	8
Metalloproteases	28–35	- Astacin-like Metalloprotease (LALPs) [29,52] - Present in the venom of different species of *Loxosceles *genus [12,13,27,51,53] - Activity upon gelatin, fibronectin, fibrinogen and entactin [18,52,53,54]	4
Hyaluronidases	41–43	- Classified as endo-beta-N-acetyl-d-hexosaminidases hydrolases [14] - Activity upon hyaluronic acid and chondroitin sulphate [13,14] - Present in the venom of different species of *Loxosceles *genus [12,13,14,24,27,51,55]	-
Serine-proteases	85–95	- Gelatinolytic activity [19] - Activated *in vitro *by trypsin [19] - Present in the venom of *L. intermedia *and *L. laeta *[27,51]	-
Serine/Cysteine protease inhibitors	N.D.	- Belongs to Serpin superfamily [27] - Identified in *Loxosceles *spp. transcriptomes and proteome [24,27,51] - May be related to coagulation processes, fibrinolysis and inflammation [51]	-
TCTP (translationally controlled tumour protein)	~46	- Identified in *Loxosceles *spp. transcriptomes [27,51] - Putative functions: Histamine releasing factor in extracellular environment; several intracellular roles such as embryonic development, cell proliferation, stabilization of microtubules [56]	-
Lectin-like	N.D.	- Putative features: carbohydrate-binding molecules; involved in extracellular matrix organization, endocytosis, complement activation, *etc*. [51]	-
Alkaline-phosphatase	N.D.	- Degrades the synthetic substrate *p-nitrophenyl phosphate*[10]	-
ATPase	N.D	- ATP hydrolysis [10]	-

N.D.: not determined. *Number of sequences deposited in PUBMED protein database.

## 3. The Rational Use of Venom Toxins as Biotechnological Tools

The idea of using venom toxins as tools for biological purposes is currently gaining acceptance worldwide, as researchers incorporate the use of novel technologies to overcome old obstacles such as low venom volumes. Technological advancement has led to better techniques for protein purification; different models for synthesis of recombinant toxins; structural views of molecular domains, binding sites or catalytic sites of molecules of interest; design of synthetic inhibitors or agonists; and finally, cellular and animal models for testing the products obtained. The use of toxins directly as a source of materials to produce medicines or similar products has been receiving much attention from the pharmaceutical industry and experts in the field of applied research. Examples of toxin-derived biomedicines derived from venoms of different animals are abundant. Venoms from snakes, perhaps the best studied example of biotechnological applications among animal venoms, with biologically active toxins in the cardiovascular system, central nervous system, membrane lipids and proteins, hemostatic system, and muscular system, have led to the discovering of several products used in the treatment of various diseases. These drugs include Captopril (blood pressure), Integrilin (acute coronary syndrome), Aggrastat (myocardial infarct and ischemia), Ancrod (stroke), Defibrase (acute cerebral infarction and angina pectoris), Hemocoagulase (hemorrhage), and Exanta (anti-coagulant). Toxin-derived products from snake venoms have also been used for diagnosis. This group of compounds includes Protac (protein C activator, diagnosis of hemostatic disorders), Reptilase (diagnosis of blood coagulation disorder) and Ecarin (diagnostic of hemostatic disorder) (for review, see [57,58]). 

Other toxin-derived medicines have been prepared from components of marine cone snail venoms, called conotoxins, which are potent ion channel modulators, and have facilitated the discovery of a novel analgesic agent named ziconide, used in the treatment of pain syndromes [59,60]. The honeybee venom toxin, called tertiapin (TPN), is an inhibitor of potassium channels, has generated TPNLQ, a variant and a potential novel model for the treatment of hypertension [61]. Exenatide (synthetic exendin-4) is a toxin-derived medicine from the venom of Gila monster lizard that stimulates the production of insulin by pancreatic cells and has the potential to treat type 2 diabetes [62,63]. Scorpion venom toxins have been studied as well, and a large number of molecules with biological activities as pain-killers, agents that control the spread of cancer, and natural insecticides can be generated. Scorpion venom, such as kurtoxin and anuroctoxin, can target specific mammalian cell ion channels and their isolation has opened possibilities for drug design in the context of neurologic and autoimmune diseases [64,65]. Other scorpion venom toxins (beta-toxins) can selectively interact with insect voltage-gated sodium channels and can be used as toxin-based pesticides [66]. Sea anemone venom toxins have been reported as potential agents for the treatment of autoimmune diseases such as multiple sclerosis, rheumatoid arthritis and type I diabetes [67]. These toxins, such as Shk, a 35-residue polypeptide toxin that is a potassium channel blocker, have proven to be very useful sources of pharmacological tools. Furthemore, the molecule’s analogs have been evaluated with regard to the development of new biopharmaceuticals for autoimmune disorders [68,69]. 

With regard to spider venoms, researchers are involved in the study of insecticidal toxins, which can be used as tools in the elaboration of environmentally safe pesticides. Notably, the venom of the Australian funnel web spider has been analyzed, with emphasis on the toxin omega-atracotoxin (ALTX) HV1, a 37-residue peptide molecule. One model proposes the use of baculoviruses to express spider toxin to act as a pesticide [59,70]. Additionally, spider venom toxins can be used as models for the development of transgenic plants expressing insecticidal toxins. One example of this situation is the case of omega-ACTH-Hvt1 toxin from the venom of *Hadronyche versuta*, which protects the tobacco plant against insects. Another rational use of spider venom toxin as a model for design of therapeutic agents involves use of the toxin from *Phoneutria nigriventer* venom as a tool for the treatment of erectile dysfunction. The toxin Tx2–6 causes an improvement in the level of nitric oxide in penile tissue in rats [71,72]. Additionally, antibacterial peptides were identified in the venom of the *Cupiennius salei *spider. These peptides appear to act as channel-forming toxins within the bacteria wall. Analogous synthetic molecules would be expected to have great potential, especially in the age of multiple-antibiotic-resistant bacteria and related threats to human health [59,73]. 

The biotechnological uses of *Loxosceles* spider venoms have received increased attention over recent years. Notably, a spider toxin-derived product (ARACHnase) was proposed for the diagnosis of lupus anticoagulant. Also, antisera produced with *Loxosceles *venom has been used as bioproducts for serum therapy after spider accidents (for more information, see [74]). Recently, several recombinant toxins from *L. intermedia, L. laeta*, *L*. *boneti*, *L .gaucho, *and *L*. *reclusa* have been described. These include members of the phospholipase-D family [32,33,34,35,36,37,39,43], members of metalloprotease/astacin family [29,52], a member of translationally controlled tumor protein family (TCTP), a hyaluronidase, a serine protease inhibitor, a venom allergen, an insecticide toxin, member of neurotoxin/Magi 3 family , and an insecticidal toxin [75]. Recombinant molecules will not only expand our knowledge of spider biology and the pathophysiology of Loxoscelism, but as we shall discuss in the next chapters, they will also provide additional molecules for biotechnological purposes [74].

## 4. Phospholipase-D

Phospholipase-D is the most studied type of molecule present in the venom from *Loxosceles* species. In the general literature, these toxins are referred to as sphingomyelinase-D, due to their first biochemical description as enzymes capable to hydrolyze sphingomyelin substrate. Based on the IUBMB recommendations, these molecules are biochemically classified as sphingomyelin phosphodiesterases D (E.C. 3.1.4.41) [5,6] Dermonecrotic toxin is a biological term widely applied by toxinologists to *Loxosceles *phospholipase-D, due to the hallmark of brown spider bites, which trigger dermonecrosis *in vivo. *Kalapothakis *et al.* [17] have organized dermonecrotic toxins of *L. intermedia *into a protein family, denoted LoxTox, by using cDNA coding sequences of several dermonecrotic/sphingomyelinase proteins from *Loxosceles intermedia*. The authors present at least six distinct groups (LoxTox 1 to 6) based on similarities among the molecules. At the present moment, Arachnoserver [30] includes 49 toxins from the *Loxosceles* genus with biological activity patterns characterized by dermonecrosis; these toxins were denoted as brown spider phospholipase-D proteins or partial sequences following the phylogenetic analyses of sicariid SMases by Bindford *et al.* [1]. 

The *Loxosceles *and *Sicarius *genera uniquely share the dermonecrotic venom toxin phospholipase D within the Haplogyne lineage. The most prospective evolutionary scenario for the origin of this enzyme is a single origin in the most recent ancestor of the Sicariidae family [76]. Phospholipases-D vary in molecular mass between species of North American *Loxosceles *(31–32 kDa), Old World species (32–33.5 kDa) and South American *Loxosceles *(32–35 kDa) [76]. Sphingomyelinase-D activity can be detected in all (36) *Loxosceles *and *Sicarius *species already tested. Binford and colleagues (2008) proposed to call this specific gene family *SicTox* towards a rational nomenclature. Based on Bayesian analyses they also resolved two clades of SMD genes, labeled α and β. Sequences in the α clade are exclusively from New World *Loxosceles *and *Loxosceles rufescens* and include published genes for which expression products have SMase D and dermonecrotic activity. The β clade includes paralogs from New World *Loxosceles* that have no, or reduced, SMase D and no dermonecrotic activity and also paralogs from *Sicarius*. In the context of structural position and proposed active sites [40], α and β clades differ only in conservation of key residues surrounding the apparent substrate binding pocket [3]. 

The pathological mechanisms of brown spider phospholipase-D have been continuously investigated, Van Meeteran [48] and Lee and Lynch [41] observed that recombinant *Loxosceles* SMaseD isoforms are able to hydrolyze lysophospholipids, generating bioactive lipid mediators such as lysophosphatidic acid (LPA). These researches extended the boundary of knowledge, which had depended upon sphingomyelin as a well-known substrate molecule. Furthermore, Lee and Lynch [41] also postulate that the term **phospholipase-D** (**PLD**) would more effectively represent the broad range of hydrolysable phospholipids than previously supposed to be applied for dermonecrotic toxins from *Loxosceles* genus [48]. Nomenclature of these toxins should be updated to account for the recent accumulation of knowledge regarding the biological and biochemical properties of these compounds.

The great interest of toxinologists in PLD proteins, to the neglect of other toxins present in the venom (most of them also enzymes or bioactive peptides), is due to the ability of these proteins to reproduce many effects of necrotic arachnidism or Loxoscelism. The PLDs from the *Loxosceles* genus are described as being responsible for several biological properties ascribed to whole venom, including the following: dermonecrosis, massive inflammatory response with neutrophil infiltration and complement activation, platelet aggregation, immunogenicity, edema and increased blood vessel wall permeability, hemolysis, renal failure, toxicity for several cultured cell types, and animal lethality [4,38,74,77]. 

Clinical investigations by Futrell [5] indicated that a dermonecrotic factor was responsible for histopathological observations resembling those of the cutaneous Arthus reaction, as observed in victims of accidents with brown spiders. Futrell [5] also reported the native toxin from *L. reclusa* (32 kDa) was an enzyme that hydrolyzes sphingomyelin and releases choline and N-acylsphingosine phosphate (or ceramide 1-phosphate). Various isoforms of phospholipase D were already reported for different species. Using SDS-PAGE analysis and chromatography methods, a range of molecular mass between 30–35 kDa was determined for PLD toxins that have hemolytic, necrotic and platelet aggregation activity, from *L. reclusa*, *L. rufescens*, *L. gaucho*, *L. laeta *and *L. intermedia *venoms [5,15,16,44,47,78,79]. Advances in proteomic studies have facilitated the description of many more PLD-related proteins in whole venom. Luciano *et al.* [80] performed two-dimensional electrophoresis and observed enriched levels of a 30-kDa molecule as well as cationic properties in *L. intermedia *whole venom, indicating the presence of several PLD-related protein spots. Furthermore, proteomic analysis of *L. gaucho *whole venom led to the identification of at least eleven PLD proteins (30–32 kDa ‘loxnecrogin’ isoforms) by Edman chemical sequencing and capillary liquid chromatography-mass spectrometry [25]. In summary, PLDs are dermonecrotic toxins that comprise a family of toxins with different related isoforms that have biological, amino acid and immunological similarities and which are found in diverse *Loxosceles* species [4,27,38,74]. This variation in phospholipase-D molecules may be due to post-translational modification and the expression of paralogous genes, since recent data demonstrate that gene duplications are frequent and that PLD genes lie in a region with high recombination within the genome [3].

Nowadays, heterologous systems based on cDNA sequences encoding mRNA transcripts from the brown spiders are a very useful tool for the production of recombinant PLD proteins (mainly in prokaryotic models). Using extracts of the venom gland, which is the tissue that is specialized for the production and secretion of venom toxins, molecular biology techniques were optimized to obtain several sequences as template for the identification, characterization and recombinant expression of PLD proteins [74]. 

At present, a new generation of molecules developed through cloning techniques still remains under investigation by researchers aiming to determine molecular and cell mechanisms of PLDs by biological approaches. *L. intermedia *LiD1 recombinant protein (31.4 kDa) is a sphingomyelinase D family molecule without dermonecrotic activity but with antigenic activity [32]. *L. laeta *recombinant protein (33 kDa) is a sphingomyelinase isoform able to degrade sphingomyelin [43]. *L. laeta *recombinant phospholipase-D generates lysophosphatidic acid and induces lysis of red blood cells [41]. Keratinocyte apoptosis was induced by recombinant PLD (SMaseD P2) from *L. intermedia *[81]. Global gene expression changes in fibroblast cells induced by PLD recombinant protein from *L. recluse* (SMD) are related to components of inflammatory response, such as human cytokines, genes involved in the glycosphingolipid metabolism pathway, and proteins known to impact transcriptional regulation [49]. Six isoforms of phospholipase-D were cloned from a cDNA library of *L. intermedia* gland venom and then expressed; they were shown to have similar toxic effects to those of native venom toxins [34,35,36,37,38]. *L. intermedia *recombinant protein (LiRecDT1, 34 kDa) displays dermonecrotic activity and was able to directly induce nephrotoxicity in mice and cultured tubular epithelial cells [42,46]. It could also induce non-complement-dependent hemolysis *in vitro* and inflammatory response using endothelial cell membrane as target [42,45]. Nephrotoxicity and hemolysis are both toxic effects that depend directly on catalytic enzyme activity. In the same way, LiRecDT2 (ABB69098), LiRecDT3 (ABB71184), LiRecDT4 (ABD91846), LiRecDT5 (ABD91847), and LiRecDT6 (ABO87656) were identified, cloned and characterized as PLD proteins with high similarity to each other based on sequence alignment; this similarity is due primarily to conserved amino acids at the catalytic site [34,35,36,37]. The results of this alignment corroborated with the crystal structure analysis of a dermonecrotic toxin [40] from *L. laeta*, which suggested there were conserved residues at the proposed catalytic site for SMase D. The recent transcriptome analysis of *L. intermedia* venom gland identified at least two clusters (annotated as PLD-related ESTs) as new possibilities for a novel PLD isoform in *L. intermedia* venom, adding a new group to the LoxTox family classification [17,27]. 

The knowledge of structural, biochemical and biological properties of PLD toxins could be employed in design studies for the development of new drugs, biopharmaceuticals, diagnostic tests and other biotechnological and industrial applications. Immunoassays using brown spider PLDs as probes have been tested [50,82] because differential diagnosis of brown spider bites can often lead to misdiagnosis [83,84]. Moreover, therapeutic serum development and vaccination have been studied to ascertain the benefits of antivenom [85,86]. Synthetic peptides designed based on PLDs toxins with specific biological/protective effects have also been utilized [87,88]. Additionally, brown spider PLDs could be employed in the development of a vaccine derived from the phospholipase-D-mutated toxin from *L. intermedia* (substitution of the Histidine12 for Alanine in the catalytic site—LiRecDT1H12A) for the immunization of people living in regions that are endemic for accidents involving *Loxosceles* spiders. This method may be useful because enzyme activity of LiRecDT1H12A is dramatically decreased and has neither hemolytic activity nor nephrotoxicity [45,46]. Another possible application for PLD is as reagent of immunodiagnostic assays for identification and quantification of phospholipase-D in the sera of patients bitten by *Loxosceles* spider because diagnosis of Loxoscelism is very controversial and is commonly based on clinical signs and symptoms [89]. Brown spider venom may be detected in hair, wound aspirates, and skin biopsy for at least seven days after inoculation [90]. 

PLD enzyme activity triggers the degradation of the cell membrane phospholipids, loss of membrane asymmetry, phosphatidylserine exposure and membrane reorganization [91,92,93]. Sphingomyelin degradation changes membrane properties, such as lipid raft organization and membrane fluidity, triggering intracellular pathways [94,95]. Phospholipid metabolites induce the release of prostaglandins, activate the complement cascade, stimulate platelet aggregation, and enhance neutrophil chemotaxis and inflammation. Brown spider PLD toxins could be used in lipid protocols for cell membrane studies related to biological effects of lipid metabolites, with emphasis on sphingolipid-derived bioactive molecules and their signaling pathways. The activity and expression of some phospholipases are increased in several human cancers, suggesting that these enzymes may have central roles in tumor development and progression [96,97]. This involvement raises the possibility of considering phospholipid metabolism as a potential target for the development of new antitumoral agents by using brown spider PLDs as a novel model for tumor cell studies. 

Further studies improving the understanding of PLD catalysis are relevant not only for comprehension of phospholipases mechanisms in basic sciences, but also for related pharmaceutical and biotechnological applications [98]. The catalytic activity of brown spider PLD plays a role in the pathological activity of this toxin and therefore cannot be dismissed as a rational target for new strategies to treat Loxoscelism. Degradation of the phospholipid head-groups by brown spider PLDs changes membrane surface potential and affects the functional properties of some cation channels. Brown spider PLDs can offer an effective pharmacological way to activate voltage-gated channels that could be useful for “channelopathy” studies [99]. Certainly, elucidation of the roles of PLDs in a variety of molecular and cell biology mechanisms might be the greatest value of brown spider PLDs as a biotechnological product, which depends on their continuous characterization with regard to the details of pathogenesis and biochemistry.

## 5. Hyaluronidase

Hyaluronidases are enzymes that mainly degrade hialuronic acid (HA), and which may have activity upon chondroitin, chondroitin sulfate (CS) and, to a limited extent, dermatan sulfate (DS) [14,100,101]. The hyaluronidases are a group of enzymes that are distributed widely throughout the animal kingdom. They were discovered through the observation that extracts of some tissues contained a “spreading factor”, which facilitated the diffusion of dyes and subcutaneous antiviral vaccines [102]. These enzymes are present in the venoms of multiple organisms, such as lizards, scorpions, spiders, bees, wasps, snakes and stingrays [103,104,105]. 

Hyaluronidases in venoms have been described as “spreading factors” due to their ability to degrade extracellular matrix components and to increase the diffusion of other toxins in tissues adjacent to the inoculation site [103]. Data from crystallography and X-ray diffraction suggested the evolutionary conservation of many poison hyaluronidases in a comparative study of several animal venoms [106,107]. Tan and Ponnudurai [108] reported that all venoms exhibit a wide range of hyaluronidase and protease activities. With regard to spider venoms, Kaiser [109] was the first to report hyaluronidase activity, from Brazilian *Lycosa raptoral* spiders, now known as *Phoneutria nigriventer *[110]. Shortly after that report, hyaluronidase activity was detected in the venom of European window spider *L. tredecimguttatus* and of the tarantula *D. hentzi *venom. This enzyme was isolated from the funnel web *A. robustus* and the tarantula *E. californicum venom* [111]. Spider venom hyaluronidases have been described more recently in *Lycosa godeffroy*, *Lympona cylindrata/murina* [110] and *Cupiennius salei*[112]. The *Hipassa* genus showed similar hyaluronidase activity to that of *H. agelenoides, H. lycosina* and *H. partita* species [110,113]. Moreover, venom obtained from *Vitalius dubius*, a spider found in southeastern Brazil, showed high levels of hyaluronidase activity [114]. With regard to necrotizing Australian spiders, hyaluronidase activity was demonstrated in *Badumna insignis*, *Loxosceles rufescens*, and *Lampona cylindrata* [12]. 

In 1973, Wright *et al.* were the first to describe hyaluronidase activity in venom of the genus *Loxosceles* [55]. This work was performed with *L. recluse* venom, and the purified enzymes, which were estimated to have molecular weights of 33 and 63 kDa by SDS-PAGE [115], exhibited activity against HA and CS types A, B, and C. The authors also showed that rabbit anti-venom inhibited the spreading effect exhibited by whole venom *in vivo* and completely inhibited hyaluronidase activity *in vitro* [55]. Young and Pincus [12], analyzing *L. recluse* venom, described hyaluronidase activity for a protein determined to be 32.5 kDa by HA-substrate SDS-PAGE [12,115]. Barbaro *et al.* [13] studied venoms from five *Loxosceles* species of medical importance in the Americas (*L. deserta, L. gaucho, L intermedia, L. laeta and L. recluse*). 

Hyaluronidase activity was detected in all species of *Loxosceles* spider venom tested by HA zymogram. All venom samples contained an enzyme with molecular weight of approximately 44 kDa, which was able to digest HA and which may contribute to the characteristic gravitational spread of the dermonecrotic lesion in patients suffering from the effects of these venoms [13,115]. da Silveira *et al.* [14] reported that zymography showed *L. intermedia *venom included hyaluronidase molecules of 41 and 43 kDa molecular weight. The activity of these enzymes is pH-dependent, with optimal activity between 6 and 8, and was able to degrade HA in rabbit skin. Pedrosa *et al.* [51] studying *L. laeta *transcriptome found transcripts with similarity to *Bos Taurus *'hyaluronidase' (gb|AAP55713.1): 4 clones and 1 cluster (LLAE0048C), representing 0.13% of the total sequence. In addition, hyaluronidase represents only 0.1% of all total toxin-encoding transcripts in the venom gland of *L. intermedia* [27]. This result may explain the difficulty associated with purification this enzyme from *Loxosceles* venoms. To obtain the recombinant hyaluronidase from *L. intermedia* venom, through the use of appropriate molecular biology techniques, an isoform was cloned and showed to have a theoretical molecular mass of about 46.1 kDa [75]. 

Hyaluronidase-mediated degradation of HA increases membrane permeability, reduces viscosity and renders tissues highly permeable to injected fluids. This degradation process is involved in bacterial pathogenesis, the spread of toxins and venoms, fertilization, and cancer progression [102]. Therefore, brown spider hyaluronidase could be used therapeutically in many fields, including orthopedics, surgery, ophthalmology, internal medicine, oncology, dermatology and gynecology [74]. There are several studies showing that hyaluronidases can be used to promote resorption of excess fluids, to increase the effectiveness of local anesthesia and to diminish tissue destruction by subcutaneous and intramuscular injection of fluids [100,102]. For example, hyaluronidase has been used to reduce the extent of tissue damage following extravasation of parental nutrition solution, electrolyte infusions, antibiotics, aminophyline, mannitol and chemotherapeutic agents, including Vinca alkaloids [116]. 

Additionally, recombinant human hyaluronidase (rHuPH20) has been used in chronic pain management, to improve systemic absorption and bioavailability of drugs [117,118,119,120]. In the context of cancer therapy, testicular hyaluronidase (HAase) has been added to drug regimens to improve drug penetration. In limited clinical studies, HAase has been used to enhance the efficacy of vinblastin in the treatment of malignant melanoma and Kaposi’s sarcoma, among other cancers [121]. Furthermore, when the level of HA decreases under conditions in which hyaluronidase activity increases, the moisture and tension of the skin are reduced, and histamine is released from mast cells [122]. Therefore, the identification and characterization of hyaluronidase inhibitors could be relevant to the development of contraceptives, as well as anti-tumor, anti-microbial, and anti-venom, anti-wrinkle, and anti-aging agents, and allergy and inflammation suppressors [14,122,123,124]. Therefore, *Loxosceles* recombinant hyaluronidases are associated with numerous potential applications [27,74,125,126].

## 6. Translationally Controlled Tumor Protein (TCTP)

*Loxosceles intermedia* TCTP protein was identified during an *L. intermedia *venom gland transcriptome study [27], although another spider TCTP had already been described from the venom gland of *Loxosceles laeta* by transcriptome analysis [51]. Proteins of the TCTP superfamily were first identified in the late eighties by research groups studying translationally regulated genes. These proteins were named *translationally controlled tumor proteins* when the discovery of human cDNA was published [127]. This name was based on the protein’s tumoral origin, a human mammary carcinoma, and on the observation that TCTP is regulated at the translational level. The translationally controlled tumor protein (TCTP), which was initially named P21, Q23 and P23 by three different groups and is also called HRF (histamine-releasing factor), represents a large family of proteins that are highly conserved and ubiquitous in eukaryotes [56,128]. 

Sequence alignment studies of TCTP sequences revealed that nearly 50% of all amino acid residues are preserved. Among species from the same genus, TCTPs are completely conserved [56]. When the TCTP sequence found in the *L. intermedia* venom gland transcriptome was compared with the one described in the venom gland of *L. laeta*, 97% similarity was observed. *L. intermedia *TCTP also presented important similarities with the other arthropod TCTPs, such as *Ixodes scapularis* and *Amblyomma americanum* from mites [27]. The scientific community’s understanding of TCTP’s biological function is growing. The compound possesses a wide range of functions, and different biochemical roles are currently being established [56,129]. 

Although TCTP participates in various biological functions, the primary physiological roles of this protein are still unknown [130]. TCTP is widely expressed in many tissues and cell types, and its protein levels are highly regulated in response to a wide range of extracellular signals and cellular conditions [56]. Interactions between TCTP and other cellular proteins have already been reported for tubulin [131], actin-F [132], the mammalian Plk [133], translation elongation factors eEF1A and eEF1Bbeta [134], Mcl-1 [135,136], TSAP6 [137], Na,K-ATPase [138], Bcl-XL [139] and Chrf [140]. Studies have already shown that TCTP is essential for embryonic development and cell proliferation in mice and *Drosophila* [141,142]. Moreover, the protein has calcium-binding activity and is capable of stabilizing microtubules, a property that may be related to a possible role of TCTP in cell cycle control, as it was also shown that TCTP interacts with a checkpoint protein (Chrf) [56,140]. 

*Loxosceles intermedia* transcriptome analysis highlighted TCTP transcript as a toxin-coding messenger due to TCTP extracellular activities already described above [27]. TCTP was described as a protein that triggers histamine release in basophil leukocytes and was therefore called ’histamine release factor’ (HRF) [128]. Then, other studies reported that TCTP presents more general ‘cytokine-like’ activity, as it also induces the production of interleukins from basophils and eosinophils [143]. TCTP itself is induced by certain cytokines and acts as a growth factor for B-cells [144]. Studies demonstrate that TCTP triggers histamine release in basophile leukocytes by mechanisms that may be dependent on or independent of the presence of IgE. It is believed that a specific TCTP receptor may participate in the process, leading to mast cell activation [56]. Although TCTP protein was found in biological fluid of asthmatic or parasitized patients and in saliva from ticks, TCTP mRNAs do not code for a signal sequence and no precursor protein has been described [56,145]. TCTP secretion from cells proceeds via an endoplasmic reticulum/Golgi-independent or non-classical pathway, probably mediated by secreted vesicles called exosomes, which have been suggested as possible pathways for non-classical secretion [137,145]. In the case of the *Loxosceles *venom gland, TCTP is secreted via holocrine secretion [27]. TCTPs have been described in gland secretions of many arthropods, such as ixodid ticks and in the venom gland of the wolf spider [146,147,148]. 

*L. intermedia* TCTP is very similar to *Dermacentor variabilis *TCTP, which is expressed in diverse tissues from the tick, including its salivary gland. When this TCTP was cloned and expressed as a recombinant protein, it was able to release histamine from a basophilic cell line [27,146]. Based on these data, it is possible to suggest that *L. intermedia* TCTP may act as a histamine release factor. The presence of a component in *L. intermedia* venom related to the histaminergic activity of venom supports with this hypothesis [149]. Recently, some authors have called attention to the role of histamine and its receptors in the development of edema, involving increased vascular permeability and vasodilatation [150], which occurs in Loxoscelism. Histamine had been described as the principal pharmacological component in the venom of the wolf spider (*Lycosa godeffroyi*) [148,151]. Proteins of the TCTP family were described to be expressed in human parasites suggesting that could be related to the survival mechanisms of parasites in the host and to the onset of pathological processes [152,153,154]. The antimalarial drug artemisin [155], probably acts on *Plasmodium* TCTP, confirming its important function in the development of pathology [153,154]. 

Recently, an increasing number of researchers have focused their attention on the cellular and extracellular activities of TCTP, as it has been implicated in the promotion of cell growth and tumorigenesis as well as in protection against apoptosis and other consequences of cell stress [56,156,157,158]. TCTP protein levels are upregulated in cancer cells and in human tumors [159,160,161]. Downregulation of TCTP has been implicated in biological models of tumor reversion [159,162], and the protein is the target of various anticancer drugs [159,163]. TCTP has been proposed as a potential cancer biomarker [160,164,165] and therapeutic target [166]. 

TCTP has enormous biotechnological potential; this toxin presents a wide range of putative applications: from a biological tool at research laboratories to clinical oncology, as a biomarker and/or a model for drug design to cancer treatment. Drugs that cause inhibition of TCTP activity resulted in tumor growth inhibition both *in vitro *and *in vivo* [159]. TCTP and its biological tools (e.g., antibodies against TCTP) can also be used in experimental oncology to study tumor cell behavior and metabolism, as well as in the screening of anticancer drugs. Still in the field of cell proliferation, TCTP and its related biological tools could also be used to study cell cycle regulation and the microtubule cytoskeleton, as well as its role in cell physiology and organelle transport. 

Calcium metabolism and signaling are other issues that could be explored using TCTP and its derived biological tools. Antiapoptotic activities were also described for TCTP: this protein potentiates MCL1 and BCL-X_L_ inhibits BAX [158]. These effects highlight TCTP as a candidate for apoptosis studies, as an apoptotic drug and as a model for anti-apoptotic reagents. Another possible application of this toxin could be its employment in allergic screening tests, due to TCTP’s histaminergic activity. Inhibitors of TCTP are putative anti-histaminic drugs and other TCTP-derived biological tools could be useful at research laboratories that study histamine release, mast cell metabolism and activation, immediate hypersensitivity reactions and the allergy process in general. Protocols that involve proliferation of B cells represent other potential applications for TCTP. TCTP secretion to the extracellular milieu is mediated by a non-classical pathway involving exosomes [137]; therefore, it is a good reagent with which to study this type of cellular secretion. TCTP has a surprising number of different functions as described here, but how these different functions might be interrelated remains to be determined [167]. Therefore the putative applications suggested herein are just the first insights into the potential uses and applications of TCTP in the field of biotechnology.

## 7. Astacin-Like Metalloproteases

Metalloproteases in *Loxosceles* venom were first characterized in *L. intermedia* venom. Feitosa *et al.* [18] described two metalloproteases, Loxolisin A (20–28 kDa, with fibronectinolytic and fibrinogenolytic activity) and Loxolisin B (32–35 kDa, with gelatinolytic activity). Zanetti *et al.* [168] purified a 30 kDa molecule with fibrinogenolytic activity from *L. intermedia* crude venom. Furthermore, da Silveira *et al.* [53] showed that venom gland extracts from brown spiders possess proteolytic activity, and this activity could be inhibited by bivalent chelators. This study proved that metalloproteases are components of *L. intermedia* and *L. laeta* venoms, and eliminated the possibility that electrostimulated venom could have been contaminated with digestive hydrolytic enzymes during extraction [53]. 

Metalloproteases were also identified as components of different *Loxosceles* species venoms, such as *L. rufescens, L. gaucho*, *L. laeta*, *L. deserta* and *L. reclusa* [12,13,51,168]. Recently, a recombinant metalloprotease from the *L. intermedia* venom gland, named LALP (*Loxosceles* astacin-like metalloprotease), was characterized as an astacin-like enzyme. This functional characterization supported previous data describing metalloproteases in *Loxosceles* venom [52]. The identification of LALP in *L. intermedia* venom was the first report in the literature of the presence of an astacin family member as an animal venom constituent. Trevisan-Silva *et al.* [29] described two new astacin-like toxin isoforms from *L. intermedia* venom (LALP2 and LALP3) and found that metalloproteases in *L. laeta* and *L. gaucho* venoms are also members of the astacin family. This study described the presence of a gene family of astacin-like toxins in three *Loxosceles* species suggesting that these molecules will be found in all South America *Loxosceles* species [29]. Astacin-like proteases are the second most commonly expressed class of toxins in the *L. intermedia* venom gland, comprising 9% of all transcripts [27]. 

The astacin family enzymes are zinc-dependent metalloproteases, which are considered as part of the metzincin superfamily [54,169]. Members from the astacin family are ubiquitous, existing more than 200 described astacins, which are found in some bacteria species and in all animal kingdoms [169,170,171,172,173]. Astacins are characterized by the zinc-binding motif (**H**EXX**H**XX**G**XX**H**EXXRXDR), which contains three histidine residues that are responsible for the complexation of zinc. Below the active site, all astacins have a methionine residue within a typical Met-turn (SX**M**X**Y),** with a tyrosine residue that might be involved in substrate fixation [54,169,174,175,176]. This protease family was named after the identification of astacin from freshwater crayfish, *Astacus astacus*. Astacin is the prototypical digestive collagenolytic enzyme of the astacin family [177,178]. Astacin family members are reported to have a wide range of functions, playing roles in digestion, in peptide and matrix molecules processing, in the activation of growth factors and in the degradation of distinct proteins [169,174,175]. 

We have little information about the biochemical and biological function of *Loxosceles* venom astacins because astacin members have distinct functions and the study of astacins from *Loxosceles* venoms is just beginning. Previous studies of *Loxosceles* metalloproteases have shown that they degrade some matrix proteins (fibronectin, fibrinogen, gelatin and entactin), but the mechanism involved in the noxious effect of the venom is until unclear [18,20,21,52]. It has been suggested that astacin toxins could be involved in gravitational spreading of dermonecrosis, in hemorrhagic disturbances observed in accidents, imperfect platelet adhesion and increased vascular permeability, which can occur near bite sites after brown spider accidents [13,29,52]. Also, astacin proteases could act as a spreading factor for other venom toxins and could serve as important agents, in the processing of other venom toxins, by cleaving inactive proteins and generating active peptides that may be involved in Loxoscelism effects [29,52]. 

Astacin-like proteases are biologically active enzymes that have potential applications in pharmaceutical studies and could be used as tools for research protocols [74]. The enzymatic activities of astacins upon different proteins highlight these molecules as useful tools in studies involving protein degradation, especially the degradation of extracellular matrix (ECM) components. Considering the physiological and pathological events related with ECM degradation, astacins can be used in protocols for medical and pharmaceutical research, such as ECM assembly and remodeling (including collagen processing and the healing process). Drug administration (as a co-adjuvant), cell membrane metabolism, embryogenesis, cellular differentiation (including stem cells), tumorigenesis and metastasis, enzymatic activation (latency and activation of zymogens), cell signaling based on proteolysis, inflammatory response and vascular permeability are other potential applications for these molecules. 

Astacins from *L. intermedia* could also be used as starting materials to design new drugs/molecules, as agonists and/or inhibitors. One possible therapeutic use of astacins from *L. intermedia *is the context of vascular diseases (acute myocardial infarction, acute ischemic stroke, thrombosed aortic aneurysms, pulmonary embolism, etc.) and as thrombolytic agents. At present, intravenously administered tissue plasminogen activator (IV-TPA) remains the only FDA-approved therapeutic agent for the treatment of ischemic stroke within three hours of symptom onset. Although intra-arterial delivery of the thrombolytic agent seems effective, various logistic constraints limit its routine use and, as yet, no lytic agent has received full regulatory approval for intra-arterial therapy [179]. Moreover, astacin inhibitors may be therapeutically useful in atherosclerosis prevention. Meprins, which are members of the astacin family, hydrolyze and inactivate several endogenous vasoactive peptides, some of which could alter various functions of cells in the arterial wall. Recent studies have shown that a meprin inhibitor suppresses the formation of atherosclerotic plaques [180]. The recombinant astacins could also be used as reagents for laboratorial tests to diagnose Loxoscelism, as well as anti-loxosceles serum production, in the treatment of envenomation.

## 8. Insecticidal Peptides

Spider venoms are functionally related to defense against predators and primarily used to paralyze and capture natural prey, especially insects [89,181,182,183]. To execute these functions, spiders developed an arsenal of insecticidal molecules in their venoms, resulting in a combinatorial peptide library of insecticidal peptides that has been improved over the course of evolution [184]. Such peptides consist of single-chain, low molecular weight molecules of 3–10 kDa, with a high number of cysteine residues that form intramolecular disulfide bridges [185,186]. Over the last decade, these peptides have been investigated extensively through identification, purification, characterization and cloning studies [23]. 

The insecticidal peptides act in the nervous system of prey or predator, causing paralysis or even death, by interacting with specific neuronal ion channels of the excitable membranes [183]. These peptides can be classified depending on their mode of action, such as effects on sodium (Na^+^), calcium (Ca^2+^), potassium (K^+^) and chloride (Cl^-^) ion channels [111,187]. Many of these peptides present a structural motif designated as an inhibitory cystine knot (ICK), and therefore these molecules are named *knottins*. The ICK motif is composed of a triple-stranded, anti-parallel β-sheet, stabilized by a cystine knot containing three disulphide bridges [188,189], which confer rigidity to the molecules in addition to a stabilization of their secondary structures and relative resistance to denaturation [190]. 

Although there are a great number of insecticidal peptides characterized in several spider species, little is known about insecticidal molecules in *Loxosceles* spiders. By studying *L. intermedia* venom, de Castro *et al.* [23] first described and characterized three isoforms of insecticidal peptides named LiTx1, LiTx2 and LiTx3 which contain ICK motif and act on specific ion channels. The chromatographic fraction containing these peptides showed potent insecticidal activity against the agricultural pests *Spodoptera *species. LiTx1 (7.4 kDa) presents some sites to possible post-translational modifications, such as N-myristoylation, protein kinase C phosphorylation, amidation and casein kinase II phosphorylation. With regard to its specificity, the study was not able to determine whether LiTx1 interacts with Na^+^ or Ca^2+^ channels. LiTx2 (7.9 kDa) and may present N-myristoylation, protein kinase C phosphorylation and amidation sites. Its specificity to ion channels was not determined. LiTx3 peptide (5.6 kDa) has also sites for N-myristoylation and protein kinase C phosphorylation. Based on bioinformatic analyses, de Castro, *et al.* hypothesized that LiTx3 may interact with Na^+^ channels. In 2006, a new isoform, LiTx4, was identified (GenBank nº DQ388598.1). 

Transcriptome analysis of the *L. intermedia* venomous gland revealed ESTs with similarity to LiTx peptides described by de Castro *et al.* [23]. LiTx3 was the most abundant sequence in the *L. intermedia* transcriptome, comprising 32% of toxin-encoding messengers. LiTx2 had a representativeness of 11% in relation to the toxin-encoding transcripts. [27]. The transcriptome analysis of *L. intermedia* venomous gland additionally revealed the presence of another class of ion channel-binding peptides. These peptides present similarity to neurotoxin Magi 3, a peptide isolated by Corzo *et al.* [26] from the venom of the *Macrothele gigas* spider. Magi 3 peptide is able to paralyze insects, although the authors did not confirm whether Magi 3 is specific for insect sodium channels or also acts on calcium channels [191]. 

The specificity of insecticidal peptides for ion channels provides an important tool to understand their dynamic activity. Ion channels are transmembrane proteins involved in the control of ion fluxes across the membrane, regulating membrane potential and ion balance. Their activity is also related to the coordination of diverse cellular functions such as excitation-contraction coupling, hormone and neurotransmitter secretion and gene expression. Thus, the comprehension of the interaction between peptide-ionic channels allows a more refined investigation of the physiological role of ion channels, as well as the determination of possible therapeutic applications [192]. 

The ability to discriminate insect ion channels confers to insecticidal peptides with considerable potential in the development of an efficient bioinsecticide for the control of economically disadvantageous pests or insect vectors of new or re-emerging disease [182,193]. Recombinant baculovirus containing the gene encoding an insecticidal peptide has been studied and tested against many insect pests, such as *Heliothis virescens* (cotton bollworm), *Laspeyresia pomonella* (codlingmoth) and *Neodiprion sertifer* (European sawfly) [183,194]. This biotechnological development could lead to alternative methods for chemical control, resulting in many benefits to the agricultural sector that will ultimately reduce economic losses.

## 9. Serine Protease Inhibitors

The control of proteases is normally achieved by the regulation of expression, secretion, activation of proenzymes and degradation. A second level of control is based on specific inhibition of activity. Despite microorganisms that produce non-proteinaceous compounds that block host proteases, the remaining all known natural protease inhibitors are proteins [195,196,197]. Among these natural protease inhibitors, the most extensively studied and described protein inhibitors of proteases are the group of serine protease inhibitors. 

Serine protease inhibitors can be classified into one of three different types, according to their structures and the mechanism of inhibition: the canonical inhibitors, the non-canonical inhibitors and the serpins. The largest group is the canonical inhibitors, which are small proteins (14 to ~200 amino acid residues) represented mainly by the Kazal, BPTI (bovine pancreatic trypsin inhibitor), potato I and STI (soybean trypsin inhibitor) families [198,199]. Non-canonical are usually found in blood-sucking organisms and are responsible for blocking the blood-clotting cascade [196]. Serpins (*ser*ine *p*rotease *in*hibitors) are large proteins (typically 350 to 500 amino acids in size), also widely distributed in nature, and are abundant in human plasma. Similar to the canonical inhibitors, serpins exhibit binding loops and interact with the target enzyme in a substrate-like manner. However, cleavage of the serpin loop by the protease leads to dramatic conformational changes in the global structure of the inhibitor [196,200,201]. 

In brown spider venom, protease inhibitors were first reported in *L. laeta* [51]. The transcriptome analysis approach, which detected 0.6% of sequences with identity to intracellular coagulation inhibitor from *Tachypleus tridentatus* and sequences with identity to serine (or cysteine) proteinase inhibitors from *Mus musculus*, *Aedes aegypti*, *Branchiostoma lanceolatum*, *Gallus gallus*, and *Boophilus microplus *. Similar results were obtained for *L. intermedia* [27], in which one transcript presented significant similarity with a serine (or cysteine) peptidase inhibitor, clade I, member 1 from *Mus musculus*. In both cases (*L. laeta* and *L. intermedia*), the sequences analyzed were similar to serine proteinase inhibitors belonging to the Serpin superfamily. 

Playing roles as potential toxins, serine protease inhibitors have been intensively described in several snake venoms, especially for those of the *Elapidae* and *Viperidae* families [202]. In these venoms, the majority of inhibitors characterized belong to the canonical type, particularly the Kunitz/BPTI inhibitors of trypsin and chymotrypsin. The peptides were typically 6–7 kDa in size and were isolated from crude venoms and studied by different methods [203,204,205,206,207,208,209,210]. The identification of this type of molecule allowed future isolation and further characterization of putative protease inhibitors, suggesting the possibility of a biotechnological application. The best example for this purpose is Textilinin-1, which is a well-known 6.7 kDa Kunitz-type serine protease inhibitor from the venom of the snake *Pseudonaja textilis *which binds and blocks certain proteases, including plasmin and trypsin [211]. The ability to reversibly inhibit plasmin has raised the possibility of using this drug as an alternative to aprotinin (Trasylol^®^), as a systemic antibleeding agent in cardiac surgery. Like aprotinin, Textilinin-1 (in equimolar concentrations) almost completely inhibits tissue plasminogenactivator-induced fibrinolysis of whole blood clots. In mouse bleeding models, Textilin-1 shows shorter time of hemostasis compared to aprotinin and appears to be a more specific plasmin inhibitor than aprotinin [210,211,212].

Despite their presence in the majority of snake venoms, serine protease inhibitors have also been described and characterized in other organisms. Zhao *et al.* [213] isolated and characterized a 60 kDa serpin from skin secretions of *Bufo andrewsi*, which was denoted as Baserpin. This protein was able to irreversibly inhibit trypsin, chymotrypsin and elastase. Serine protease inhibitors are also present in spider venoms, particularly in the venom of tarantulas (*Ornithoctonus huwena* and *Ornithoctonus hainana*). The prototypic molecule in tarantula venom is HWTX-XI, 6.1 kDa peptide from *Ornithoctonus huwena* venom, which belongs to the Kunitz-type family of serine protease inhibitors. Just like Kunitz-type toxins in snake venoms, HWTX-XI is considered to be a bi-functional toxin because it is a strong trypsin inhibitor as well as a weak Kv1.1 potassium channel blocker [214].

Zhao *et al.* [213] isolated and characterized a 60 kDa serpin from skin secretions of *Bufo andrewsi*, which was denoted as Baserpin. This protein was able to irreversibly inhibit trypsin, chymotrypsin and elastase. The considerations above represent just a few insights concerning serine protease inhibitors uses and applications. The great importance of proteases in numerous different biological processes and the large number of protease inhibitors described suggest their strong biotechnological potential.

## 10. Conclusion

Research in brown spider venom toxins has increased over recent years, but the challenges and opportunities are enormous. To move the field forward, scientists must have access to the biodiversity of spiders within their countries. Different *Loxosceles* genus spider species are reported to inhabit every continent [5,6,8], and bureaucracy related to the capture of spiders should not be a hindrance to researchers on toxinology area. Official collaborations with groups based where brown spiders are endemic will facilitate access to their venom. 

Another difficulty in working with *Loxosceles* venoms is the fact that the volume of venom is minute (just microliters, containing a few micrograms of protein, as previously discussed). This makes work difficult for researchers that use crude venom in their experiments. To overcome this difficulty, works can collect venom from hundreds, or even, thousands, of spiders during specific periods of the year when there is an abundance of spiders and store the venom under appropriate conditions (*i.e*., lyophilized or in solutions at −80 °C) [18]. Alternatively, brown spiders could be captured from the wild and kept individually (because they kill one another) under laboratory conditions, using insect larvae as food and with periodic hydration via water-soaked cotton balls, with venom collected as necessary. 

Another technical solution for venom production is the standardization of long-term primary culture of secretory cells from the venom gland and the production of venom *in vitro*. The culture of secretory cells from different venomous animals has shown promising results, and represents a good system with which to obtain toxins without capturing animals from the wild and without the related ecological disturbances. To date, several groups have reported expertise on this topic, and have established protocols for the primary culture of secretory cells. Examples include those from the venom glands of *Crotalus durissus terrificus* and *Bothrops jararaca* snakes [215,216], as well as those from the venom glands of the *Phoneutria nigriventer* spider [217]. Such protocols ensure that sufficient amounts of native toxins are produced and secreted for culture medium and used for technical purposes after purification. Unfortunately, for *Loxosceles* venom gland cells, there are no reports to date of successful primary cultures of secretory cells. This situation represents a rational challenge for the future regarding the acquisition of sufficient amounts of native molecules. Finally, the venom of *Loxosceles* species is commercially available, as is the case for *L. deserta* (Sigma, St. Louis, USA). 

The cDNA library construction of *L. intermedia* venom gland [35], transcriptome analysis [27,51] and the cloning and synthesis of several recombinant toxins [29,32,33,34,35,36,37,39,43,52] is helping to elucidate the biology of *Loxosceles* genus and opening possibilities for biotechnology applications. Recombinant toxins have been expressed in bacteria, simple organisms that are easy to manipulate and cheap to work with; unfortunately these do not generate co- and post-translational modifications such as disulphide bonds and protein glycosylations. Certain recombinant molecules are expressed in their unfolded form, have incorrected conformations, are water insoluble, and have no biological function. 

With regard to phospholipase-D family members, these recombinant toxins purified from bacteria have biological functions compatible with those described for native toxins. For native toxins, it was already very well demonstrated that inflammatory response with cytokines release is induced at the bite site, and lipid content might be relevant for tissue damage [218,219]. These recombinant toxins induce dermonecrosis, platelet aggregation, increased vessel permeability, deep inflammatory responses, and phospholipase-D activity [34,35,36,37]. On the other hand, a great number of brown spider venom recombinant toxins synthesized by bacteria are water-insoluble and have no biological function. To surpass this technical obstacle, insoluble toxins can be refolded by methods of protein refolding [220], but the final concentration of refolded toxins obtained is generally not enough for biotechnological uses. 

Alternatively, toxins can be synthesized using other expression models, such as the yeast *Pichia pastoris* [221], an organism that has subcellular organelles as endoplasmic reticulum and Golgi apparatus. This yeast is able to perform co- and post-translational modifications of proteins. For *Loxosceles* toxins, preliminary experiments are underway [75], but a frequent problem to be overcome is the hyperglycosylation of secreted proteins, which alters the biological functions of the toxins. Expression in systems of insect cells, such as *Drosophila* Schneider cells, is a possible alternative method [222] because it is a eukaryotic expression system, in which proteins undergo post-translational modifications. 

For *Loxosceles* toxins, again, experiments are just beginning and results are preliminary [75], but they can provide secreted toxins that are correctly folded and, in the near future, may be used as tools for biological evaluations. Baculovirus vector for protein expression in insect and mammalian system is also feasible [223], but we do not have information on *Loxosceles* molecules produced using this technique. Finally, the mammalian expression system is a rational alternative for expression of correctly folded recombinant proteins. Mammalian cells have the capacity for proper protein folding and assembly, as well as co- and post-translational modifications [224]. Currently, there are no data on *Loxosceles* venom toxins obtained using this system. However, because this model is a viable method for recombinant proteins of therapeutic use, scientists are expected to explore this system in the future. 

The advancement of *Loxosceles* venom toxin research will also involve techniques from proteomic analysis. These techniques generally have high sensitivity and accuracy and normally use low venom concentration for analysis. To date, at least two works have been completed addressing this topic. By using proteomics methodologies, such as bi-dimensional electrophoresis, N-terminal amino acid sequencing and mass spectrometry, eleven isoforms for phospholipase-D toxin were identified in *L. gaucho* venom [25]. In addition, through mass spectrometry analysis using *L. intermedia* crude venom, 39 proteins were identified, and putative effects for envenomation were discussed [24]. The use of combinatorial data from proteomic and molecular biology techniques, such as mass spectrometry, transcriptome analysis and cDNA library constructions, will open possibilities for the discovery of novel toxins in complex venoms [225]. 

Additionally, in the near future, the biotechnological use of *Loxosceles* toxins could provide information related to the tridimensional structure of identified toxins, through crystallography and X-ray diffraction and/or nuclear magnetic resonance for soluble toxins [59]. Findings in these areas will bring insight related to the molecular structure of toxins and will be very important for the discovery of catalytic sites, sites that interact with natural substrates or ligands, and from such data, synthetic ligands, analogs, or inhibitors could be designed for biotechnological purposes. 

Regarding *Loxosceles* spider venom toxins, a recombinant phospholipase-D from *L. laeta* was analyzed by crystallography and X-ray diffraction. The data collected allowed description of the amino acid residues involved in catalysis and metal ion coordination important for sphingomyelinase activity [226]. Experiments using other isoforms of phospholipase-D from *L. intermedia* venom (LiRecDT1, LiRecDT2, LiRecDT6, GFP-LiRecDT1, and LiRecDT1H12A, with a mutation on the catalytic site, [46]) are currently being conducted using crystallography and X-ray diffraction. Additionally, other *Loxosceles* recombinant toxins (enzymes and peptides) could be evaluated and represent potential biological tools in a wide range of fields.
